# A Wearable Silent Text Input System Using EMG and Piezoelectric Sensors

**DOI:** 10.3390/s25082624

**Published:** 2025-04-21

**Authors:** John S. Kang, Kee S. Moon, Sung Q. Lee, Nicholas Satterlee, Xiaowei Zuo

**Affiliations:** Department of Mechanical Engineering, San Diego State University, San Diego, CA 92182, USA; jkang4@sdsu.edu (J.S.K.); sqlee@sdsu.edu (S.Q.L.); nsatterlee0532@sdsu.edu (N.S.); xzuo1800@sdsu.edu (X.Z.)

**Keywords:** silent text input, biomedical signal processing, wearable biomedical sensors, machine learning, sensor fusion, speech disability, human–computer-interface

## Abstract

This paper introduces a wearable silent text input system designed to capture text input through silent speech, without generating audible sound. The system integrates Electromyography (EMG) and piezoelectric lead zirconate titanate (PZT) sensors in a miniaturized form that can be comfortably attached to the chin, making it both comfortable to wear and esthetically pleasing. The EMG sensor records muscle activity linked to specific tongue and jaw movements, while the PZT sensor measures the minute vibrations and pressure changes in the chin skin caused by silent speech. Data from both sensors are analyzed to capture the timing and intensity of the silent speech signals, allowing the extraction of key features in both time and frequency domain. Several machine learning (ML) models, including both feature-based and non-feature-based approaches commonly used for classification tasks, are employed and compared to detect and classify subtle variations in sensor signals associated with individual alphabet letters. To evaluate and compare the ML models, EMG and PZT signals for the eight most frequently used English letters are collected across one hundred trials each. Results showed that non-feature-based models, particularly the Fea-Shot Learning with fused EMG and PZT signals, achieved the highest accuracy (95.63%) and F1-score (95.62%). The proposed system’s accuracy and real-time performance make it promising for silent text input and assistive communication applications.

## 1. Introduction

Traditional text input and speech recognition systems rely on audio signals, which are easily detected and processed by microphones. While successful in many settings, these systems are unsuitable for situations where vocalization must be minimized or avoided entirely. Examples include high-security areas, noisy environments, or for individuals who cannot speak due to various medical conditions. Silent speech technologies aim to provide an alternative by detecting the physiological signals associated with speech production, such as muscle activity in and around the tongue.

Among the various technologies used to capture speech-related signals, Electromyography (EMG) sensors have been widely explored for detecting muscle activity associated with speech production. While several studies have demonstrated the potential of using EMG for silent speech recognition (SSR), most of these systems are not wearable. This is due to the large size of the systems, limitations in wireless and real-time data transmission, or the requirement to attach electrodes to the face or neck, which can make the systems uncomfortable or impractical for prolonged use.

Many SSR systems use complex, multi-channel EMG signal acquisition, which depends on bulky, non-wearable hardware. These systems typically require multiple electrodes to be attached to the face or neck, making them uncomfortable and impractical for long-term use. Meltzner et al. emphasized that their SSR system required face- and neck-worn sensors arranged in multi-point geometries to record high-fidelity EMG signals [1]. Ratnovsky et al. noted that while their EMG-based speech recognition approach showed high accuracy, the reliance on multiple facial electrodes made it impractical for real-world use [2]. Song et al. utilized a high-density sEMG system with a 64-channel electrode array for silent speech decoding, achieving high accuracy but at the cost of wearability [3]. Zhu et al. highlighted the need for a large number of electrodes to capture neuromuscular activity comprehensively, which increases system complexity and reduces its portability [4]. Additionally, Rodríguez-Tapia et al. reviewed the state of EMG signal processing and emphasized that existing myoelectric interfaces often require specialized laboratory setups, making them impractical for daily use [5]. Wu et al. also discussed how multi-channel EEG and sEMG-based SSR systems need complex amplifiers and signal processing units, further contributing to their non-wearable nature [6]. Srisuwan, Prukpattaranont et al. compared various classifiers for EMG-based speech recognition and found that while electrode placement on the face and neck improved recognition accuracy, it also caused discomfort and limited usability [7]. Lee et al. reviewed various biosignal-based speech recognition systems and noted that while deep learning techniques have improved recognition accuracy, there remains a gap in developing practical, wearable SSR devices [8]. Bu et al. suggested that reducing the number of electrodes using differential EMG signals could be a step toward more user-friendly systems [9]. Jou et al. proposed phoneme-based acoustic models incorporating specifically designed surface EMG feature extraction methods to facilitate continuous speech recognition [10].

Professor Tanja Schultz and her collaborators have made significant contributions to the field of SSR by exploring the integration of EMG signals for speech decoding and addressing challenges related to sensor placement and system practicality. In one study, Schultz et al. developed a wearable silent speech interface using surface EMG sensors, highlighting the trade-off between recognition accuracy and wearability [11]. Another study by Schultz’s team focused on optimizing EMG-based SSR systems for mobile applications, emphasizing the importance of reducing the number of electrodes to improve comfort and usability [12]. Their research continues to bridge the gap between high-accuracy SSR systems and practical, real-world implementations.

Piezoelectric lead zirconate titanate (PZT) transducers have been used for SSR in a few studies. Wang et al. proposed a piezoelectric MEMS-based unvoiced speech-recognition sensor that captures oral airflow patterns for non-acoustic SSR, enhancing privacy and usability in noisy environments [13]. Jung et al. used flexible piezoelectric sensors to detect speech-related vibrations without acoustic signals, offering robust performance even in noisy environments, and advanced this concept by integrating machine learning to enhance recognition accuracy for low-volume and silent speech [14].

Real-time processing of sensor data remains a challenge due to the high computational demands of feature extraction and classification algorithms. Gonzalez-Lopez et al. highlighted that most current SSR systems have only been validated in laboratory settings, with little progress in wireless and real-time applications [15]. Hofe et al. discussed the difficulty of integrating real-time processing into wearable devices, as silent speech interfaces often require high-resolution EMG data that is difficult to transmit wirelessly without latency issues [16]. Zhu et al. noted that high-density sEMG electrodes generate large volumes of data, requiring substantial computational power for real-time processing [4]. Wu et al. combined EEG and sEMG signals for improved recognition, but the need for complex data synchronization and wireless transmission was identified as a major limitation [6].

While several studies have demonstrated the potential of EMG-based SSR, challenges related to system size, computational burden, wireless data transmission, and electrode placement continue to hinder its adoption as a truly wearable technology. To address these limitations, this study introduces a miniaturized, wearable silent mouth movement monitoring system equipped with a high-sensitivity EMG sensor, which can be attached under the chin to capture muscle activity associated with specific tongue and jaw movements. Additionally, the system integrates a piezoelectric (PZT) sensor to detect minute vibrations and pressure changes during silent speech. To enable real-time and wireless data transmission, the system features an integrated Bluetooth Low Energy (BLE) module and an on-board signal processing unit, enhancing its practicality as a fully wearable silent speech recognition device.

This study also employs machine learning (ML) algorithms to classify signals from the two sensors and recognize subtle variations corresponding to different alphabet letters. The ML models are trained using only a limited dataset to evaluate their feasibility for real-time training and prediction. By integrating ML techniques with a wearable silent mouth movement monitoring system, this research aims to develop a practical and accurate silent text input system. The potential applications of this technology include assistive communication devices, silent communication solutions for secure environments, and immersive input methods for gaming and virtual reality.

Section 2 introduces the wearable mouth movement monitoring system and provides details on the experimental data—EMG and PZT signals for the top eight most frequently used English letters. Section 3 presents the ML-based data processing methods used to classify the eight silent speech letters. Section 4 presents the experimental results, followed by Section 5, which discusses the findings. The paper concludes with Section 6, summarizing the main outcomes and implications of the study.

## 2. Wearable Mouth Movement Monitoring System

### 2.1. Experimental Setup

**Figure 1:**
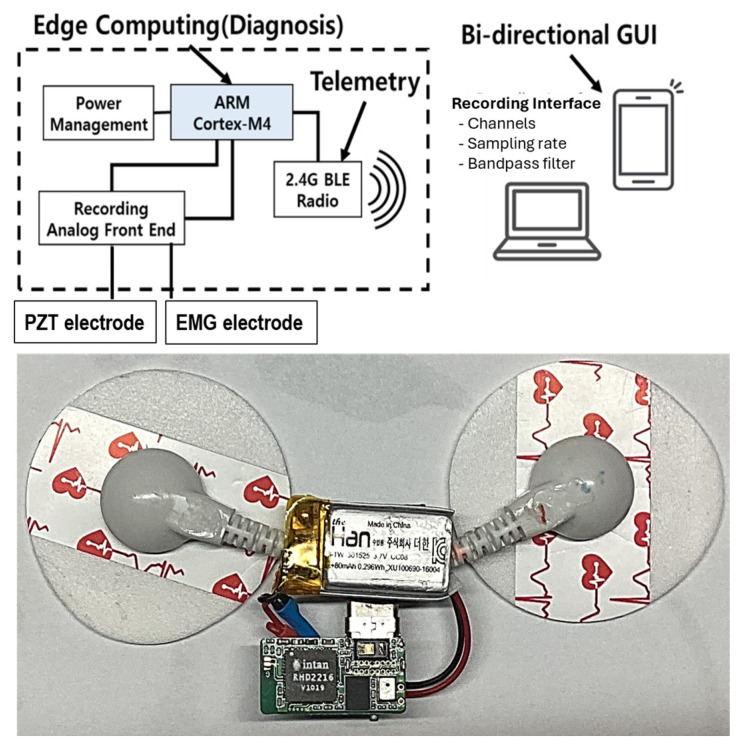
The circuit diagram of the wireless wearable mouth movement monitoring system.

This study introduces a novel EMG-PZT system [17] designed for real-time monitoring and digital identification of mouth motion activities (Figure 1). Our wearable, wireless system combines biopotential sensors to concurrently capture tongue muscle activity and jaw movement using the EMG sensor, while detecting minute vibrations and pressure changes in the chin skin using the PZT sensor. To optimize performance, the system features an on-board signal processing circuit that enables edge computing, reducing the volume of data transmitted through the wireless communication module. The processed data are then sent to an external computer system, where they undergo further analysis for feature extraction and classification. A comprehensive description of the system’s sensor components can be found in Table 1.

Precise electrode placement on the human chin is critical for the success of this experiment, as targeting specific chin regions ensures optimal data acquisition. As previously emphasized, a biopotential transducer is used to convert chin muscle movement into analog electrical signals, while a thin piezoelectric plate detects jaw motion via the chin. Signal acquisition is facilitated by Intan Technologies’ digital electrophysiology interface chips (Los Angeles, CA, USA), which offer a 4 kHz sampling rate per channel. The RHD2216 chip, a low-power 16-channel differential amplifier integrated with a 16-bit ADC, is employed to record both EMG and PZT signals.

The wearable sensor enables wireless communication through Bluetooth Low Energy (BLE), transmitting the acquired signals to a personal computer (PC) for subsequent signal processing and classification using MATLAB 2024a (Mathworks Inc., Natick, MA, USA). The system supports real-time signal acquisition, amplification, filtering, digitization, and wireless transmission. The nRF52832 System-on-Chip (SoC) from Nordic Semiconductor (Nordic Semiconductor, Trondheim, Norway) was chosen for its computational power and wireless capabilities, offering low power consumption and flexibility. In addition to two commercially available adhesive patches, a custom-designed wearable sensor was developed and fabricated specifically to serve as the EMG-PZT monitor for this experiment.

**Figure 2:**
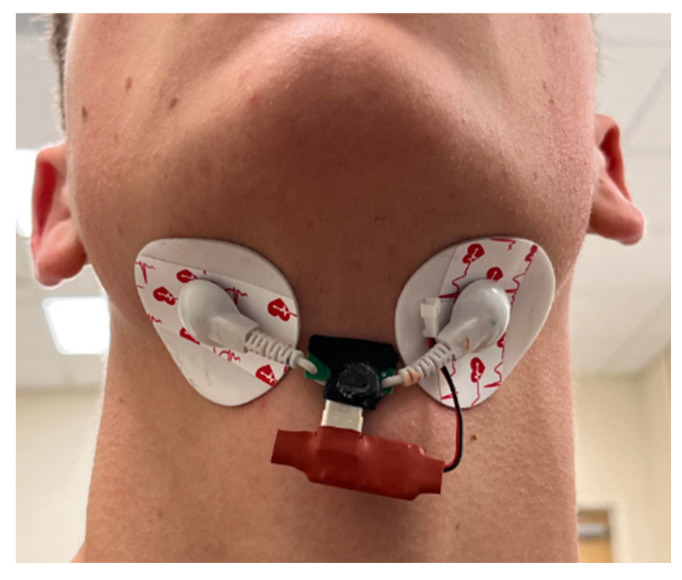
The placement of the EMG-PZT sensor under the chin.

The electrodes of the EMG sensor and the thin PZT plate are placed under the chin, as shown in Figure 2, to detect tongue muscle activity, jaw movement, and vibrations changes during silent speech. A sensor placed beneath the chin detects the complex muscular activity involved in tongue and jaw movements. This area is crucial for speech, as the suprahyoid muscles—specifically the digastric, mylohyoid, geniohyoid, and stylohyoid—directly govern these motions [18]. These muscles, situated superior to the hyoid bone (a horseshoe-shaped bone acting as a tongue anchor), coordinate to regulate the hyoid’s position, which is vital for both tongue movement and swallowing. As a result, the sensor’s location enables the acquisition of sensitive and distinct signals that reflect tongue and jaw movements, facilitating the detection of muscle activation during silent articulation of alphabet letters.

### 2.2. Experimental Data

To evaluate the performance of the wireless wearable mouth movement monitoring system and ML approach proposed in this study, we recorded sensor signals corresponding to eight letters, listed in Table 2, which represent the most frequently used letters in the English language [19]. Each letter was pronounced without sound while sensor data were collected in real time. A total of 100 trials were conducted for each letter, yielding 100 sets of sensor data per letter. To minimize motion artifacts, data were collected under static conditions, excluding any physical movement. Ensuring proper electrode–skin contact is critical for optimal data acquisition. Maintaining stable electrode placement throughout the recording period is essential, as secure contact guarantees the highest quality signal recordings.

**Figure 3:**
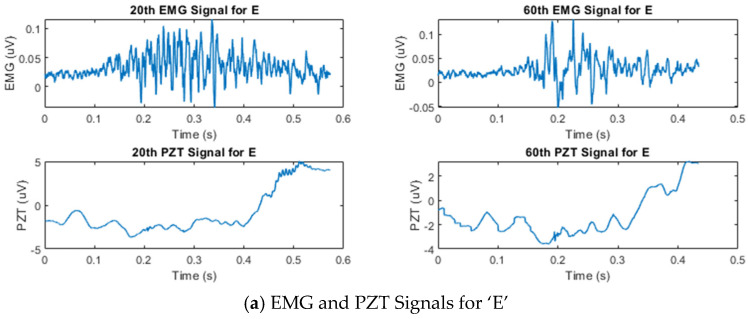

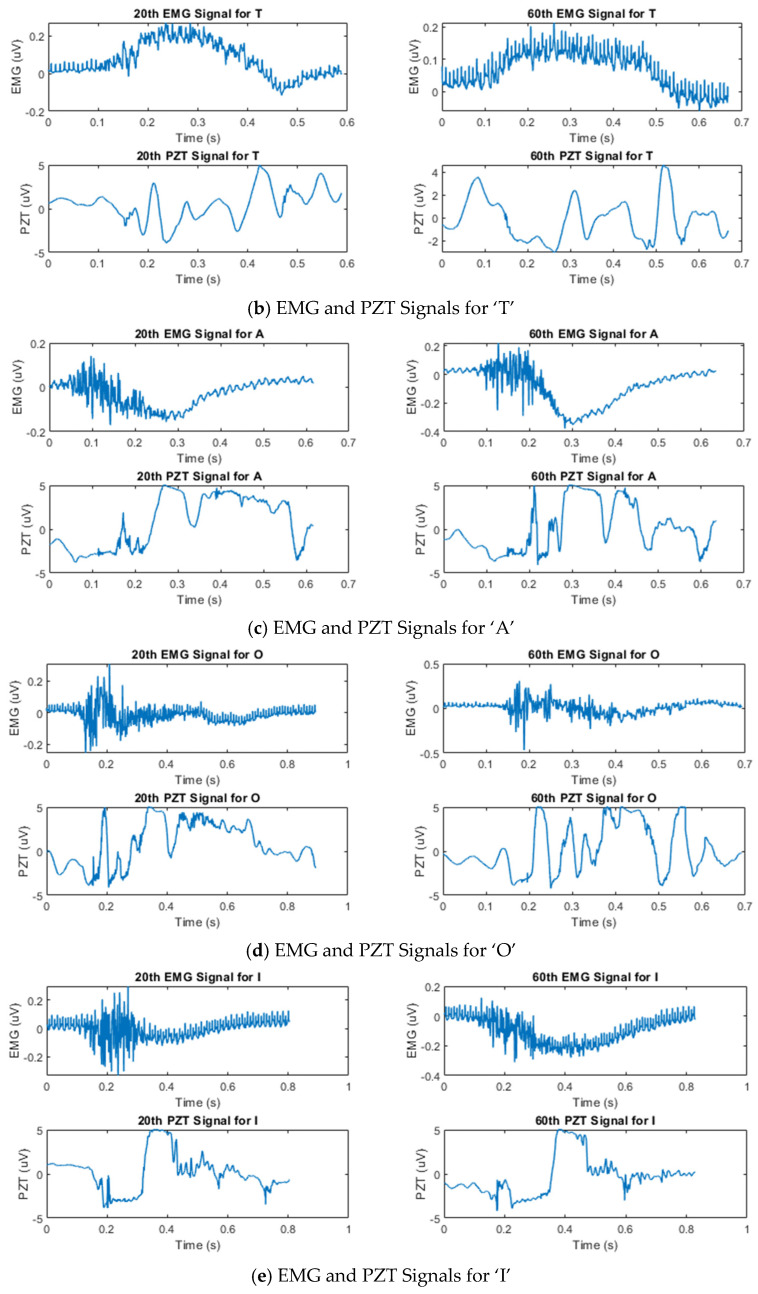

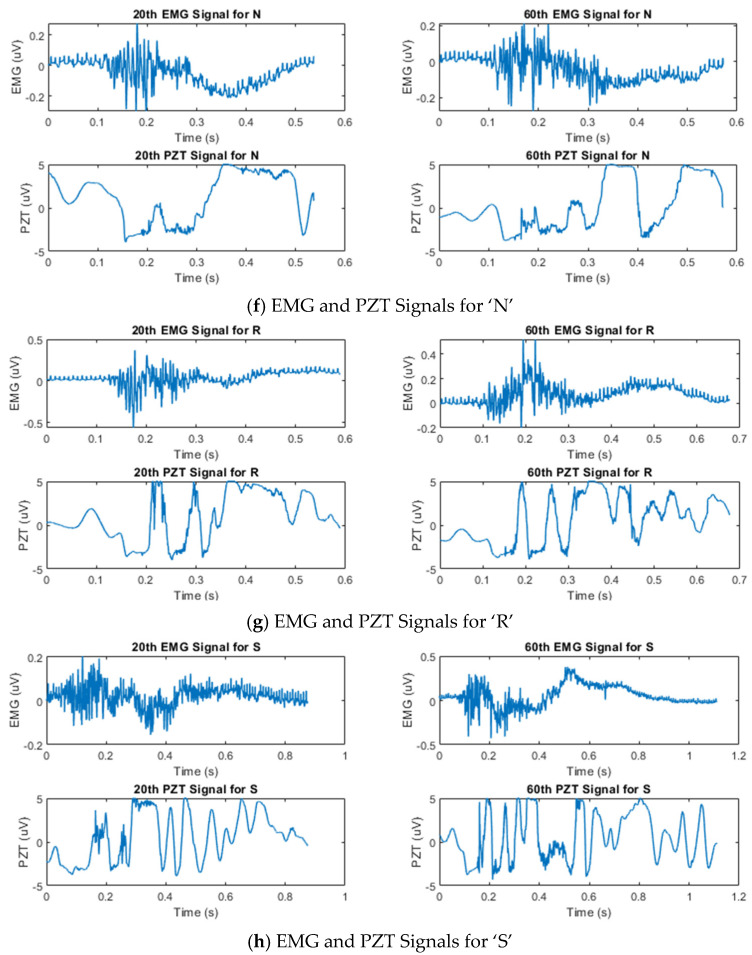
Example of synchronized EMG and PZT signals acquired during the 20th and 60th silent speech trials for each letter, illustrating their time and amplitude characteristics.

Figure 3 shows the temporal amplitude characteristics of the EMG and PZT signals acquired from the experiments. Signals from two distinct trials—the 20th and 60th trials—are presented to demonstrate the variations between them.

### 2.3. Feature Definition

To quantify the SSR signal activities, several features commonly used in various SSR studies are extracted. Directly measurable features from the raw signal include the mean absolute value, zero-crossing rate, waveform length, root mean square (RMS), and variance of the signal [20,21]. Features derived from Fourier-transformed data include dominant frequency, mean frequency, median frequency, and frequency centroid [1,20]. The descriptions of all features extracted from the SSR signals are provided in Table 3.

### 2.4. Feature Selection

High-dimensional input can impose substantial computational demands during ML model training. To address this challenge, Recursive Feature Elimination (RFE) is employed to identify an optimal subset of features, thereby reducing dimensionality [21]. RFE is a robust feature selection technique that iteratively eliminates less important attributes while building a model using the remaining features. It evaluates model accuracy to determine which attributes or combinations of attributes contribute most significantly to predicting the target variable.

The RFE process begins with the full set of features, repeatedly constructing a model, ranking features by their importance, and removing the least significant feature in each iteration until only the most critical feature remains. This backward elimination approach is particularly advantageous for high-dimensional datasets, as it systematically reduces complexity while maintaining predictive performance. By identifying the optimal feature subset, RFE not only enhances model accuracy but also improves interpretability, reduces computational costs, and mitigates the risk of overfitting.

The features selected after the RFE process were waveform length, signal RMS, frequency centroid, and mean frequency.

## 3. Silent Speech Classification Machine Learning

We evaluated several machine learning (ML) models, including both feature-based and non-feature-based approaches, to classify sensor signals corresponding to eight letters. The feature-based models were trained using the features defined in Section 2.3, while the non-feature-based models were directly provided with raw voltage readings as time-series data. These non-feature-based models automatically extract and learn relevant features through convolution operations, enabling them to identify meaningful patterns directly from the sensor signals without requiring explicit feature engineering. To enhance model performance and reduce the risk of overfitting, data augmentation techniques were applied to the training dataset, ensuring greater variability.

### 3.1. Data Augmentation for Feature-Based ML

To enhance the diversity of the training dataset and mitigate the risk of overfitting, data augmentation techniques were employed. For feature-based ML models, augmentation was applied at the feature level rather than on raw signal data. Instead of generating additional voltage readings through signal-level augmentation, the dataset was expanded directly within the feature space using the Synthetic Minority Over-sampling Technique (SMOTE) [22], as these engineered features serve as the primary inputs to the feature-based models.

**Figure 4:**
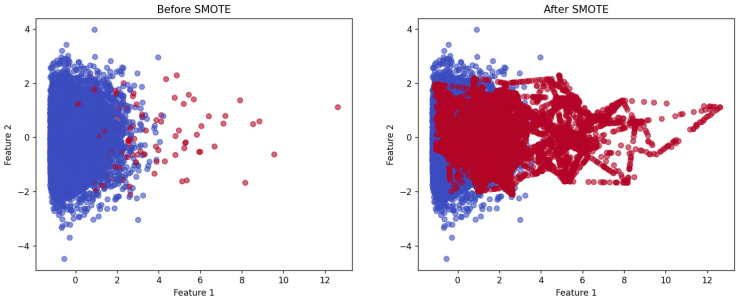
Example of feature-level data augmentation in 2D feature space.

Figure 4 illustrates the application of SMOTE in a two-dimensional feature space. The left plot shows the initial class imbalance, where the majority class (blue points) significantly outnumbers the minority class (red points). The right plot demonstrates the effect of the SMOTE, where synthetic samples are generated by interpolating between existing minority class instances, resulting in a more balanced class distribution. Unlike simple duplication, this method produces informative synthetic samples that better capture the underlying distribution of the minority class.

As a result, the augmented dataset provides a more representative training set, thereby enhancing the learning performance and generalization capability of the feature-based models. This augmentation is applied dynamically during training, contributing to the development of a more robust and adaptable model.

### 3.2. Data Augmentation for CNN

For CNN models, data augmentation was performed directly on the raw signals, as these constitute the primary input for such model architecture. This augmentation was previously demonstrated in [23]. The types of augmentation were selected to mimic scenarios that might be encountered during raw signal data collection including noise intrusion, voltage shifts, and recording errors. These scenarios are considered over long periods (i.e., applied to the entire sample) and over short periods (i.e., applied to a portion of the sample). Entire sample augmentations included adding a random slope to the sample, shifting the sample in the x-direction, and shifting the sample in the y-direction. The list of augmentations and their descriptions is displayed in Table 4. All augmentations are applied in random order to increase variability. This ensures that the augmentations are applied efficiently and in a randomized manner during training, contributing to a more robust and adaptable model.

### 3.3. Feature-Based Machine Learning Methods

For the feature-based approach, three classifiers were evaluated: Support Vector Machines (SVMs), Decision Trees (DTs), and Random Forests (RFs). These models were selected for their diverse classification paradigms. SVMs are margin-based classifiers that seek to maximize the decision boundary between different classes, demonstrating strong performance in high-dimensional spaces [24]. DTs utilize a rule-based strategy to recursively partition the input space based on feature thresholds [25]. RFs, as ensemble methods, aggregate predictions from multiple decision trees constructed on random subsets of data and features, offering enhanced robustness to overfitting and noise [26].

ML models are often sensitive to their initial configuration, which is governed by a set of hyperparameters. These hyperparameters play a critical role in shaping the model’s learning behavior and overall performance. For example, in SVM, the choice of kernel function—such as radial basis function, linear, or polynomial—substantially influences the transformation of input data into higher-dimensional feature spaces, thereby affecting classification accuracy [24].

For feature-based ML models, hyperparameter tuning was conducted using a standard grid search strategy to optimize model performance [27]. The search spanned a wide range of hyperparameter values, typically across several orders of magnitude (e.g., 0.01, 0.1, 1, 10, 100), to ensure a thorough exploration of the parameter space. The optimal hyperparameter configuration for each model was determined based on the combination that achieved the highest classification accuracy on the validation set.

### 3.4. Convolutional Neural Networks

We used Convolutional Neural Networks (CNNs) primarily because CNNs are designed to automatically extract and learn features directly from raw input data. This makes CNNs particularly well suited for sequential or time-series data like ours. CNN architectures have also been known to generalize well compared to traditional models, consistently achieving state-of-the-art performance in tasks where raw data are high-dimensional and unstructured.

For CNN models, network architecture was adjusted to enhance performance. The number of layers in the network was incrementally increased until further increases no longer resulted in decreased losses. Pooling layers were incorporated for dimensionality reduction, with adaptive 1D pooling selected for its simplicity and flexibility.

**Figure 5:**
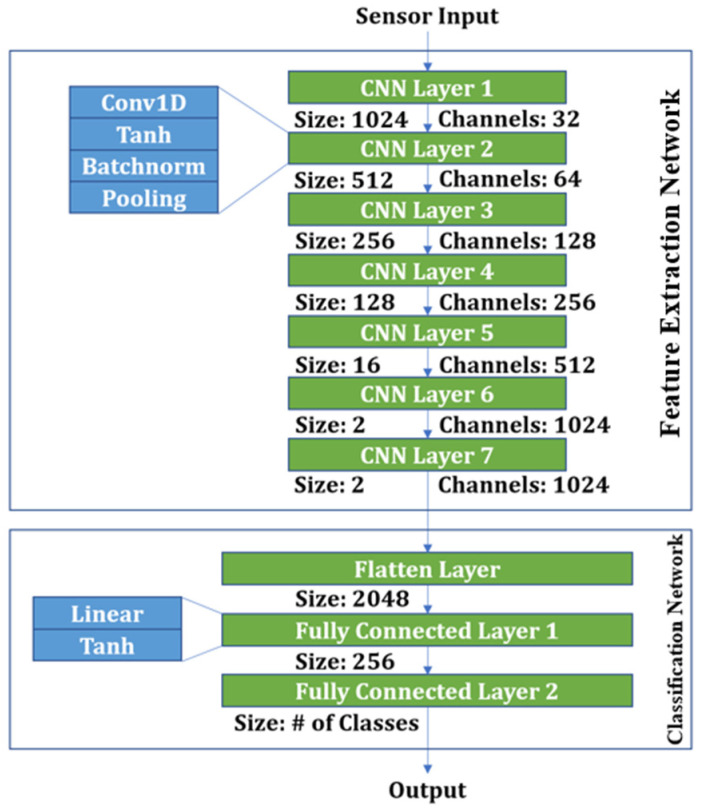
CNN architecture.

The CNN architecture is composed of two primary components: the Feature Extraction Network (FEN) and the Classification Network (CN), as shown in Figure 5. The FEN features a seven-layer structure, with the number of layers determined through experimental optimization. Each layer comprises a 1D convolution operation (Conv1D), Tanh activation function (Tanh), batch normalization (BatchNorm), and pooling [23]. The output from the FEN is flattened and then passed through two consecutive fully connected layers in the CN, reducing the dimensionality to a 256-dimensional feature vector, which is used as the input for classification tasks [23].

### 3.5. Siamese Neural Network

Few-shot learning is an ML paradigm designed to train models capable of making accurate predictions or classifications with minimal labeled data. This approach is particularly advantageous in fields where obtaining large datasets is challenging. One effective few-shot ML model is the Siamese Neural Network (SNN), which classifies new inputs by comparing them to a set of reference samples from each class. Instead of requiring a large training dataset, SNNs operate by computing similarity metrics between input and reference samples, allowing classification based on proximity in the feature space.

During training, the model parameters are optimized to form distinct clusters in the feature space, with reference samples positioned at the center of each cluster. In the testing phase, an incoming sample is compared against these fixed clusters, and classification is performed based on similarity metrics. A key advantage of SNNs is their architectural flexibility, as they are not constrained to a specific network design. This adaptability makes them highly applicable to SSR, effectively addressing the data scarcity issue commonly encountered in SSR research.

**Figure 6:**
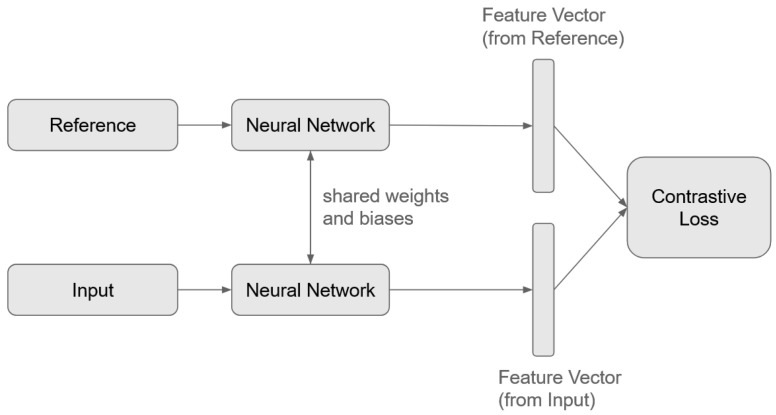
Diagram for Siamese Neural Network.

SNNs consist of neural networks trained to generate a similarity metric between input samples. Typically, an SNN is composed of two identical CNNs that share weights and biases, enabling them to extract comparable features from different input signals. Unlike traditional classification models, where the output corresponds to the likelihood of belonging to a specific class, SNNs compute distance or similarity scores between pairs of inputs. This is achieved through a contrastive loss function, which determines the similarity or dissimilarity between a provided reference sample and the input sample. Reference samples, which are pre-defined input vectors, serve as class representations within the feature space. Figure 6 illustrates the structure of an SNN, demonstrating how the model processes input pairs to compute similarity metrics.

To address the challenge of limited training data in SSR, the SNN approach with contrastive loss [28] was employed, which has been shown to outperform traditional classifiers, such as SoftMax, in data-constrained scenarios [23,29]. During the few-shot training, reference samples were first selected from the experimental dataset and passed through the network to generate feature vectors, termed reference feature vectors. Subsequently, training samples, randomly chosen from the experimental and augmented datasets, were processed by the network to create their respective feature vectors. The training process involved comparing the feature vectors of these samples to the reference feature vectors. A cosine similarity loss function was employed to guide the optimization, minimizing the distance between feature vectors of samples belonging to the same class while maximizing the distance between those of different classes. This process ensures that the network learns positive and negative representations for accurate defect classification [28].

### 3.6. Sensor Fusion

Multi-sensor systems are expected to surpass single-sensor systems in performance by integrating data from multiple sources, thereby enhancing accuracy, reliability, and robustness in identifying and interpreting complex situations. By combining diverse sensor inputs, these systems mitigate the limitations of individual sensors and offer a more comprehensive representation of the monitored environment. However, effectively fusing sensor data in a meaningful and computationally efficient manner remains a key challenge.

**Figure 7:**
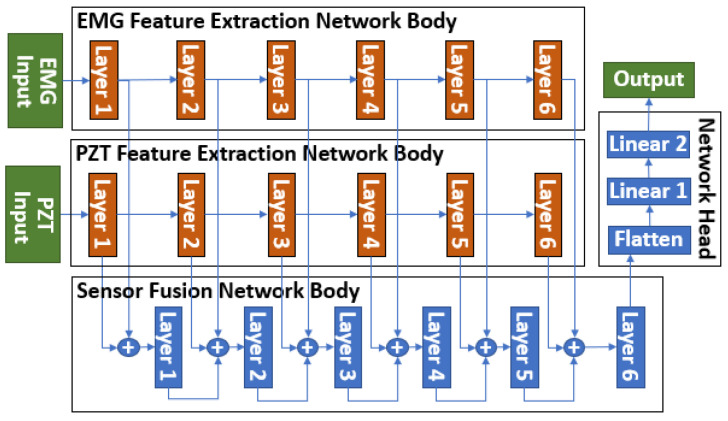
PMLDF architecture with two sensor inputs.

To address this issue, we employed a novel sensor fusion method, the Parallel Multi-Layer Data Fusion (PMLDF) approach, to integrate EMG and PZT sensor signals. Figure 7 illustrates the PMLDF architecture, where data from two sensors are processed through parallel Feature Extraction Networks, each designed to extract sensor-specific features independently. These features are then passed to a central Sensor Fusion Network, which fuses the extracted representations across multiple layers. The architecture is scalable, allowing additional sensors to be incorporated by introducing corresponding Feature Extraction Networks and connecting their outputs to the Sensor Fusion Network.

Each layer in the Feature Extraction Networks and the Sensor Fusion Network consisted of a 1D convolutional layer, followed by the hyperbolic tangent(tanh) activation function, batch normalization, and pooling layers [23]. To accommodate variations in sensor sampling rates, adaptive pooling was applied at the end of each layer, ensuring robustness to different sensor configurations. The fused output from the Sensor Fusion Network was then passed to the Network Head, where classification was performed using Artificial Neural Network (ANN) or SNN.

To maximize performance, network hyperparameters were systematically tuned. The number of layers was progressively increased until further additions no longer resulted in reduced loss. Pooling layers were incorporated for dimensionality reduction, with adaptive 1D pooling chosen for its simplicity and flexibility. Although static max pooling layers were also considered, no significant performance differences were observed between the two approaches. The learning rate was carefully optimized, as excessively small or large values led to prolonged convergence times. To facilitate efficient training, a feature encoding scheduler was applied with a step size of 10 epochs and a gamma value of 0.992, gradually reducing the learning rate during training to improve convergence stability. The patience parameter, which determines how long training continues without improvement before termination, was systematically increased in increments of 50 epochs, starting at 50 epochs. No further accuracy improvements were observed beyond 150 epochs, indicating an optimal stopping point for training.

## 4. Results

This chapter presents the results of ML experiments conducted to classify silent speech letters using the EMG and PZT sensor data. The primary objectives of the experiments were to evaluate the performance of various ML models and to investigate the influence of sensor modalities, features, and sensor fusion strategies on classification accuracy. A summary of the model training scenarios is provided in Table 5.

Scenarios 1–3 employed feature-based ML methods, including SVM, RF, and DT. Initially, models were trained separately using EMG-derived and PZT-derived features to assess the individual contribution of each sensor modality. Subsequently, the models were trained using a combined feature set comprising both EMG and PZT features. In the following scenarios (4–5), the features were optimized using the feature selection method described in Section 3.1, and the models were retrained using each signal individually as well as the combined EMG and PZT signals.

The experimental dataset was partitioned into 60% for training, 20% for validation, and 20% for testing. To enhance variability and improve model generalization, data augmentation—described in Section 3.1—was applied exclusively to the training set. We have evaluated both accuracies and F1-scores achieved across each scenario [30].

Feature-based ML models were trained using the features defined Section 2.3, extracted from each EMG or PZT signal of each letter. The dataset was divided into 60% for training, 20% for validation, and 20% for testing. The following models were evaluated: SVM, FR, and DT.

Table 6 presents the prediction Accuracy and F1-score of three feature-based machine learning models—SVM, RF, and DT—using different combinations of EMG and PZT signal features as training inputs. The table highlights the impact of both all feature inputs and selected feature inputs on classification performance.

When using only EMG features, SVM achieved the highest performance (Accuracy: 80.83%, F1: 81.19%) compared to RF and DT, which exhibited moderate (RF: Accuracy 71.67%, F1 72.62%) and lower (DT: Accuracy 50.42%, F1 48.52%) scores, respectively. In contrast, models trained solely on PZT features showed improved performance for RF (Accuracy: 79.58%, F1: 79.42%), outperforming SVM (Accuracy: 77.08%, F1: 76.84%) and DT (Accuracy: 69.58%, F1: 69.69%).

Combining both EMG and PZT features led to a significant performance boost, particularly for RF, which attained the highest accuracy (91.25%) and F1-score (91.44%) among all configurations. SVM also benefited from the combined features (Accuracy: 87.08%, F1: 86.87%), while DT’s performance remained comparatively lower (Accuracy: 68.33%, F1: 67.50%).

Feature selection further influenced model effectiveness. Notably, using selected EMG and PZT features together yielded the best results for DT (Accuracy: 83.33%, F1: 83.08%) and maintained high performance for SVM (Accuracy: 90.83%, F1: 90.98%) and RF (Accuracy: 88.33%, F1: 88.06%). This indicates that strategic feature selection not only reduces input dimensionality but can also enhance model generalizability and accuracy.

Overall, RF consistently showed superior performance when utilizing combined or selected features, while SVM performed well with EMG data and benefited from multimodal integration. DT, although generally underperforming, showed notable improvement when applied to selected feature sets.

Table 7 summarizes the classification performance of non-feature-based machine learning models—CNN and SNN—trained on raw EMG, raw PZT, and combined raw EMG + PZT signal inputs. The results are reported in terms of prediction accuracy and F1-score.

When trained solely on raw EMG data, both CNN and SNN achieved comparable performance, with CNN slightly outperforming SNN (Accuracy: 88.75% vs. 88.13%; F1-Score: 88.45% vs. 87.65%). In contrast, models trained on raw PZT signals exhibited a notable increase in performance. SNN achieved the highest accuracy (94.38%) and F1-score (94.37%) among single-modality inputs, while CNN also performed well (Accuracy: 93.13%, F1-Score: 93.02%), indicating that PZT signals contain highly discriminative information for the classification task.

The integration of both raw EMG and raw PZT inputs resulted in the best overall performance for both models. SNN slightly outperformed CNN, achieving an accuracy of 95.63% and an F1-score of 95.62%, compared to CNN’s accuracy of 95.00% and F1-score of 94.95%. This suggests that multimodal signal fusion provides complementary information that significantly enhances model performance.

Overall, SNN demonstrated slightly superior performance over CNN across all input configurations, particularly with PZT and multimodal inputs. These findings highlight the effectiveness of non-feature-based models in leveraging raw physiological signals for high-accuracy classification.

## 5. Discussion

This study explored the efficacy of ML models in classifying silent speech motions based on the EMG and PZT signals obtained using the wearable mouth movement monitoring system. The focus was on comparing various ML methods, as well as investigating the potential role of sensor fusion in improving classification accuracy. The findings provide important insights into the performance of various models and highlight both the strengths and limitations of different approaches.

The experimental results indicate that among feature-based models, RF consistently achieved the highest classification of accuracy and F1-score when using a combination of EMG and PZT features (Accuracy: 91.25%, F1: 91.44%). This suggests that RF is highly effective at capturing complex patterns when both sensor modalities are utilized. In contrast, SVM, while also benefiting from the combined features (Accuracy: 87.08%, F1: 86.87%), showed slightly lower performance compared to RF, indicating that it may be less robust in handling multimodal data. The DT model consistently underperformed compared to both RF and SVM, likely due to its relatively simple decision boundaries, which are insufficient to capture the complex relationships within multimodal features.

When analyzing individual sensor modalities, EMG features led to higher performance for SVM (Accuracy: 80.83%, F1: 81.19%) compared to PZT features (Accuracy: 77.08%, F1: 76.84%). This suggests that SVM is more suitable for processing EMG data, potentially due to its ability to handle high-dimensional feature spaces effectively. In contrast, RF demonstrated improved performance with PZT data (Accuracy: 79.58%, F1: 79.42%), reflecting its strength in handling low-frequency input data.

Interestingly, the application of feature selection positively impacted model performance. Combining selected EMG and PZT features yielded the best results for DT (Accuracy: 83.33%, F1: 83.08%) and maintained high performance for both SVM (Accuracy: 90.83%, F1: 90.98%) and RF (Accuracy: 88.33%, F1: 88.06%). This indicates that reducing the dimensionality of the input space can enhance model generalization, particularly for models prone to overfitting. Moreover, the effectiveness of selected features highlights the importance of feature engineering in optimizing classification performance, especially when computational efficiency is a concern.

The non-feature-based models—CNN and SNN—demonstrated superior performance compared to feature-based approaches. Notably, the SNN model achieved the highest overall accuracy and F1-score when using combined raw EMG and PZT inputs (Accuracy: 95.63%, F1: 95.62%), slightly outperforming CNN (Accuracy: 95.00%, F1: 94.95%). This finding highlights the potential of SNN in capturing temporal dependencies and signal dynamics inherent in physiological data of EMG and PZT signals. The superior performance of raw EMG and PZT data aligns with the notion that non-linear neural networks, such as CNN and SNN, are adept at directly processing raw signals without the need for feature extraction.

**Figure 8:**
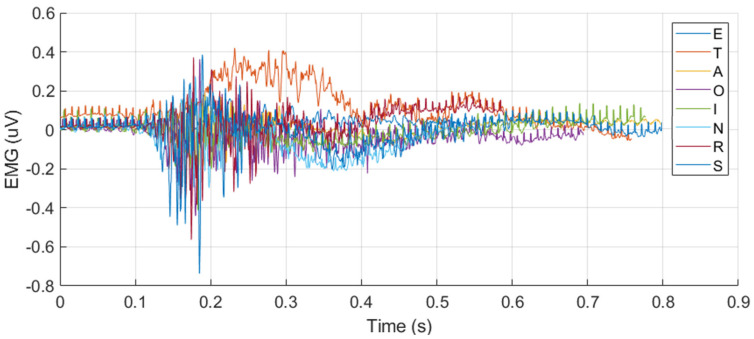

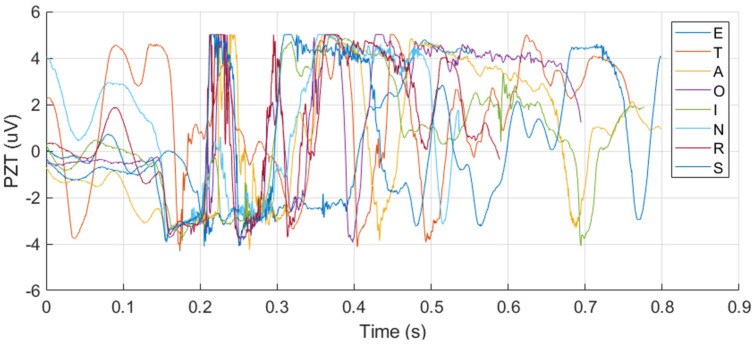
The overlapped EMG and PZT signals for the eight letters, illustrating their discriminative characteristics across different letters.

Among single-sensor inputs, PZT signals provided better classification outcomes than EMG signals for both CNN (Accuracy: 93.13%, F1: 93.02%) and SNN (Accuracy: 94.38%, F1: 94.37%). This result suggests that PZT signals contain more discriminative information for silent speech classification, as shown in Figure 8, possibly due to their higher sensitivity to subtle muscle movements compared to EMG.

Comparing feature-based and non-feature-based models reveals that non-feature-based approaches (CNN and SNN) outperform traditional ML models (SVM, RF, and DT) across all scenarios. The primary reason for this discrepancy is the ability of deep learning models to automatically extract relevant features from raw data, capturing complex signal patterns that may be overlooked in manually engineered feature sets. Moreover, the integration of both raw EMG and PZT signals consistently improved performance across all models, emphasizing the importance of multimodal data fusion for silent speech classification.

## 6. Conclusions

This study explored the use of a wearable mouth movement monitoring system, designed to capture and analyze silent speech through a combination of Electromyography (EMG) and Piezoelectric (PZT) sensors. The system was engineered for real-time wireless monitoring of tongue muscle activity and jaw movement, while simultaneously detecting subtle vibrations and pressure changes in the chin skin. The integration of these sensors into a single, wearable, wireless device presents a significant step forward in silent speech recognition technology.

The wearable system’s design is based on a custom-built EMG-PZT sensor, which is capable of acquiring both muscle and jaw movement data, transmitted wirelessly via Bluetooth Low Energy (BLE). The system includes an on-board signal processing circuit, allowing for edge computing and reducing data transmission volume, thus improving efficiency and battery life. The processed data are then transmitted to an external computer for further classification and analysis. The compact, efficient nature of this system makes it ideal for real-time, portable applications in silent speech monitoring.

In addition to the hardware’s capabilities, the study also evaluated machine learning (ML) models to classify the eight letters from the sensor data captured while articulating the letters without sound. Our results demonstrate that non-feature-based ML models—Convolutional Neural Networks (CNN) and Siamese Neural Networks (SNN)—trained directly on raw sensor signals significantly outperform traditional feature-based models—Support Vector Machines (SVMs), Random Forests (RFs), and Decision Trees (DTs)—in terms of accuracy and F1-score. Also, the sensor fusion worked effectively for both feature-based and non-feature-based ML models. Among these, SNN demonstrated the highest classification performance when sensor fusion is applied, achieving an Accuracy of 95.63% and an F1-score of 95.62%.

The wearable EMG-PZT monitoring system represents a promising approach for advancing silent text input technologies. Its real-time data acquisition, coupled with powerful wireless communication and on-board signal processing, enables practical applications in environments where traditional speech recognition may not be feasible. This study also contributes to the field of silent speech recognition by systematically comparing multiple ML approaches and highlighting the benefits of multimodal signal fusion. The findings of this study highlight the potential of this wearable system not only for silent speech input but also for broader applications in speech therapy, rehabilitation, and communication aids for individuals with speech impairments. Future work could focus on optimizing the system’s performance through improved sensor calibration, advanced feature extraction techniques, and further refinement of ML models, with an eye toward expanding its applicability and reliability in real-world scenarios.

Despite the promising results, this study has some limitations. The relatively small dataset size (eight letters) may limit the generalizability of the findings. Future work should expand the dataset to include more letters, users, and pronunciation variations. Additionally, exploring transfer learning and domain adaptation techniques could enhance model robustness across diverse user populations. Managing real-world noise is another challenge, as background noise can mask leak signals and reduce detection accuracy. Incorporating adaptive noise filtering techniques could improve resilience against diverse and variable noise sources.

## 7. Patent

The following patent is partially resulting from the work reported in this manuscript: Moon, K.S., Lee, S.Q. An Interactive Health-Monitoring Platform for Wearable Wireless Sensor Systems. PCT/US20/51136.

## Figures and Tables

**Table 1:** The specifications of the wireless wearable sensor.

Specification	Description	Value
Power source	Rechargeable battery	65 mAh/charging
Data transmission	BLE 5.0	1 M bps in 2 m
EMG electrodes	Disposable Ag/AgCl standard, pre-gelled and self-adhesive	(20 × 20) mm
PZT plate	PZT-coated metal plate	10 mm diameter
Front-end circuit	Intan Tech Chip (RHD2216)	10 mV, 16 bit, 16 ch
On-board CPU	ARM Cortex M4	4096 Hz/ch sampling rate
Wireless circuit	NRF 52X	2.4 GHz, BLE 5.0

**Table 2:** A silent speech experimental protocol.

Experiment #	Motion
1	“E” motion
2	“T” motion
3	“A” motion
4	“O” motion
5	“I” motion
6	“N” motion
7	“R” motion
8	“S” motion

**Table 3:** The descriptions of the features.

No.	Feature	Unit	Description
1	Mean Absolute Value	mV	MAV is the average of the absolute values of the signal over the recorded interval, calculated as $\frac{1}{T}\int_{t_{0}}^{t_{0}+T}\left|y\left(t\right)\right|dt=\frac{1}{N}\overset{N}{\underset{i=1}{\sum }}\left|y\left(i\right)\right|$, where $y\left(t\right)$ is the signal value in millivolts, $t_{0}$ is the start time of the recording in microseconds, *T* is the duration interval in µs, and *N* is the number of sampled data points within the duration interval.
2	Root Mean Square	mV	RMS is the square root of the average of the squared values of a signal, calculated as $\sqrt{\frac{1}{T}\int_{t_{0}}^{t_{0}+T}y{\left(t\right)}^{2}dt}=\sqrt{\frac{1}{N}\overset{N}{\underset{i=1}{\sum }}y{\left(i\right)}^{2}}$.
3	Variance	mV^2^	Variance is the dispersion of a signal value from the mean, calculated as $\frac{1}{T}\int_{t_{0}}^{t_{0}+T}{\left[y\left(t\right)-\mu \right]}^{2}dt=\frac{1}{N}\overset{N}{\underset{i=1}{\sum }}{\left[y\left(i\right)-\mu \right]}^{2}$, where *μ* is the mean value of the signal.
4	Waveform Length	Unitless	Waveform length is the total accumulated change in the signal over the recorded interval, calculated as $\int_{t_{0}}^{t_{0}+T}\left|y\left(t_{i+1}\right)-y\left(t_{i}\right)\right|dt=\overset{N-1}{\underset{i=1}{\sum }}\left|y\left(i+1\right)-y\left(i\right)\right|$.
5	Zero-Crossing Rate	Unitless	ZCR is the number of times the signal crosses the zero axis, calculated as $\overset{N-1}{\underset{i=1}{\sum }}\left|y\left(i\right)y\left(i+1\right)<0\right|$.
6	Dominant Frequency	Hz	The dominant frequency is the frequency component that has the highest amplitude in the signal spectrum.
7	Mean Frequency	Hz	The mean frequency is the weighted average of all frequency components in the signal spectrum, calculated as $\frac{\sum_{i=1}^{N}\left|y\left(i\right)\right|}{\sum_{i=1}^{N}\left|y\left(i\right)\right|}$.
8	Median Frequency	Hz	The median frequency is the frequency that divides the power spectrum into two equal parts.
9	Frequency Centroid	Hz	This is the center of mass of the frequency spectrum, weighted by magnitude.

**Table 4:** A list of augmentations with their examples and descriptions.

Augment Type	Description	Equation
Hang	A random datapoint overwrites a random consecutive sequence of subsequent datapoints.	$x^{\prime }\left(i+n\right)=x\left(i\right)\;for\;0<n<j\;$ where $x^{\prime }$ is the output, x is the input, i is random start, j is random end, and n is the number of samples.
Guass	A Gaussian filter with random kernel is applied to a random consecutive sequence of datapoints.	$x^{\prime }\left(i\right)=\overset{k}{\underset{j=-k}{\sum }}x\left(i+j\right)\cdot G\left(j\right)$ where G is Gaussian kernel, k is the kernel size.
Noise	Noise is added to a random consecutive sequence of data points scaled to the standard deviation of the data.	$x^{\prime }\left(i\right)=x\left(i\right)+N\cdot std\left(i\right)$ where $N\;\in \left[0,1\right]$, and std is the standard deviation operator.
Down-sample	A random consecutive sequence of datapoints is down-sampled between 1 and 25% of the input using linear interpolation, then up-sampled back to the original number of points.	$x'\left(i\right)=up\left(down\left(x\left(i\right)\right)\right)$ where *down* is the down-sample operator and *up* is the up-sample operator.
Slope	A linear slope is added to the entire dataset with the maximum delta Y less than 10 times the standard deviation of the data.	$x^{\prime }\left(i\right)=x\left(i\right)+\frac{slope}{n}\cdot i$ where $slope\;\in \left[0,10\cdot std\left(x\right)\right]$.
Shift X	The entire dataset is shifted randomly in the positive or negative x-direction with a maximum shift of 5%.	$x^{\prime }\left(i\right)=x\left(i+s\right)\;for\;i+s<m$ where s is random shift, and m is the signal length.
Shift Y	The entire dataset is shifted randomly in the positive or negative y-direction with a maximum shift of 5%.	$x^{\prime }\left(i\right)=x\left(i\right)+s$ where $s\in \left[0.95\cdot \Delta y,\;1.05\cdot \Delta y\right]$, $\Delta y=\max\left(x\right)-\min\left(x\right)$, and *s* is random shift.
Scale	The entire dataset is scaled randomly between 95% and 105%.	$x^{\prime }\left(i\right)=x\left(i\right)*sc$ where $sc\in \left[0.95,\;1.05\right]$.

**Table 5:** ML training scenarios.

Category	Scenario #	Input	Model
Feature-Based ML	1	EMG Features	SVM/FR/DT
	2	PZT Features	SVM/FR/DT
	3	EMG Features + PZT Features	SVM/FR/DT
	4	Optimized EMG Features	SVM/FR/DT
	5	Optimized PZT Features	SVM/FR/DT
	6	Optimized EMG Features + PZT Features	SVM/FR/DT
Non-Feature-Based ML	7	Raw EMG	CNN/SNN
	8	Raw PZT	CNN/SNN
	9	Raw EMG + Raw PZT (PMLDF)	CNN/SNN

**Table 6:** Prediction accuracy (F1-number) of feature-based machine learning models.

Training Inputs	Accuracy (%)			F1-Score (%)		
	SVM	RF	DT	SVM	RF	DT
EMG Features	80.83	71.67	50.42	81.19	72.62	48.52
PZT Features	77.08	79.58	69.58	76.84	79.42	69.69
EMG Features + PZT Features	87.08	91.25	68.33	86.87	91.44	67.50
Optimized EMG Features	82.50	79.17	72.08	82.69	79.29	72.13
Optimized PZT Features	74.58	76.25	62.08	74.14	76.48	62.33
Optimized EMG Features + PZT Features	90.83	88.33	83.33	90.98	88.06	83.08

**Table 7:** Prediction accuracy of non-feature-based machine learning models.

Training Inputs	Accuracy (%)		F1-Score (%)	
	CNN	SNN	CNN	SNN
Raw EMG	88.75	88.13	88.45	87.65
Raw PZT	93.13	94.38	93.02	94.37
Raw EMG + Raw PZT (PMLDF)	95.00	95.63	94.95	95.62

## Data Availability

The original data presented in the study are openly available in Google Drive at https://drive.google.com/drive/folders/1cLKid5QQI12D8D-sZ2hQ2D2wi0q9lOGn?usp=sharing (accessed on 15 March 2025).

## References

[1] Meltzner G.S., Heaton J.T., Deng Y., De Luca G., Roy S.H., Kline J.C. (2018). Development of sEMG sensors and algorithms for silent speech recognition. J. Neural Eng..

[2] Ratnovsky A., Malayev S., Ratnovsky S., Naftali S., Rabin N. (2023). EMG-based speech recognition using dimensionality reduction methods. J. Ambient Intell. Humaniz. Comput..

[3] Song R., Zhang X., Chen X., Chen X., Chen X., Yang S., Yin E. (2023). Decoding silent speech from high-density surface electromyographic data using transformer. Biomed. Signal Process. Control.

[4] Zhu M., Zhang H., Wang X., Wang X., Yang Z., Wang C., Samuel O.W., Chen S., Li G. (2021). Towards optimizing electrode configurations for silent speech recognition based on high-density surface electromyography. J. Neural Eng..

[5] Rodríguez-Tapia B., Soto I., Martínez D.M., Arballo N.C. (2020). Myoelectric Interfaces and Related Applications: Current State of EMG Signal Processing–A Systematic Review. IEEE Access.

[6] Wu R., Lu Z., Guan X., Zhang M., You W., Li G. Silent Speech Recognition based on sEMG and EEG Signals. Proceedings of the 2021 China Automation Congress (CAC).

[7] Srisuwan N., Prukpattaranont P., Limsakul C. (2020). Comparison of Classifiers for EMG based Speech Recognition. J. Phys. Conf. Ser..

[8] Lee W., Seong J.J., Ozlu B., Shim B.S., Marakhimov A., Lee S. (2021). Biosignal Sensors and Deep Learning-Based Speech Recognition: A Review. Sensors.

[9] Bu N., Tsuji T., Arita J., Ohga M. Phoneme Classification for Speech Synthesiser using Differential EMG Signals between Muscles. Proceedings of the 2005 IEEE Engineering in Medicine and Biology 27th Annual Conference.

[10] Jou S., Schultz T., Walliczek M., Kraft F., Waibel A. Towards continuous speech recognition using surface electromyography. Proceedings of the INTERSPEECH 2006.

[11] Wand M., Schultz T. Session-independent EMG-based Speech Recognition. Proceedings of the International Conference on Bio-Inspired Systems and Signal Processing (BIOSIGNALS-2011).

[12] Wand M., Schultz T. Towards real-life application of EMG-based speech recognition by using unsupervised adaptation. Proceedings of the INTERSPEECH 2014.

[13] Wang Q., Ruan T., Xu Q., Shi Y., Yang B., Liu J. Piezoelectric Mems Unvoiced Speech-Recognition Sensor Based on Oral Airflow. Proceedings of the 2022 IEEE 35th International Conference on Micro Electro Mechanical Systems Conference (MEMS).

[14] Jung Y., Hong S., Wang H., Han J.H., Xuan Trung P., Park H., Kim J., Kang S., Yoo C., Lee J. (2019). Flexible Piezoelectric Acoustic Sensors and Machine Learning for Speech Processing. Adv. Mater..

[15] Gonzalez-Lopez J.A., Gomez-Alanis A., Doñas J.M.M., Pérez-Córdoba J.L., Gomez A.M. (2020). Silent Speech Interfaces for Speech Restoration: A Review. IEEE Access.

[16] Hofe R., Ell S.R., Fagan M.J., Gilbert J.M., Green P.D., Moore R.K., Rybchenko S.I. (2013). Small-vocabulary speech recognition using a silent speech interface based on magnetic sensing. Speech Commun..

[17] Moon K., Lee S.Q., Youm W. (2024). Interactive Health-Monitoring Platform for Wearable Wireless Sensor System. U.S. Patent.

[18] Karunaharamoorthy A. (2024). Suprahyoid Muscles. Kenhub. https://www.kenhub.com/en/library/anatomy/suprahyoid-muscles.

[19] Solso R.L., King J.F. (1976). Frequency and versatility of letters in the English language. Behav. Res. Methods Instrum..

[20] Wu J., Zhang Y., Xie L., Yan Y., Zhang X., Liu S., An X., Yin E., Ming D. (2022). A novel silent speech recognition approach based on parallel inception convolutional neural network and Mel frequency spectral coefficient. Front. Neurorobot..

[21] Zhou S., Li T., Li Y. (2023). Recursive Feature Elimination Based Feature Selection in Modulation Classification for MIMO Systems. Chin. J. Electron..

[22] Chawla N.V., Bowyer K.W., Hall L.O., Kegelmeyer W.P. (2002). SMOTE: Synthetic minority over-sampling technique. J. Artif. Intell. Res..

[23] Moon K.S., Kang J.S., Lee S.Q., Thompson J., Satterlee N. (2024). Wireless Mouth Motion Recognition System Based on EEG-EMG Sensors for Severe Speech Impairments. Sensors.

[24] Ghaddar B., Naoum-Sawaya J. (2018). High dimensional data classification and feature selection using support vector machines. Eur. J. Oper. Res..

[25] Mienye I.D., Jere N. (2024). A Survey of Decision Trees: Concepts, Algorithms, and Applications. IEEE Access.

[26] Breiman L. (2016). Random Forests. Int. J. Adv. Comput. Sci. Appl..

[27] Liashchynskyi P., Liashchynskyi P. (2019). Grid Search, Random Search, Genetic Algorithm: A Big Comparison for NAS. arXiv.

[28] van der Spoel E., Rozing M.P., Houwing-Duistermaat J.J., Eline Slagboom P., Beekman M., de Craen A.J.M., Westendorp R.G.J., van Heemst D. Siamese Neural Networks for One-Shot Image Recognition. Proceedings of the 32 nd International Conference on Machine Learning.

[29] Liu B., Cao Y., Lin Y., Li Q., Zhang Z., Long M., Hu H. (2020). Negative Margin Matters: Understanding Margin in Few-Shot Classification. Computer Vision–ECCV 2020: 16th European Conference, Glasgow, UK, 23–28 August 2020.

[30] Grandini M., Bagli E., Visani G. (2020). Metrics for Multi-Class Classification: An Overview. arXiv.

